# Relação Metabólica e Inflamatória entre Covid-19 e Não-HDL-C

**DOI:** 10.36660/abc.20230304

**Published:** 2023-06-16

**Authors:** Renato Jorge Alves

**Affiliations:** 1 Irmandade da Santa Casa de Misericórdia de São Paulo São Paulo SP Brasil Irmandade da Santa Casa de Misericórdia de São Paulo, São Paulo, SP – Brasil; 2 Faculdade de Ciências Médicas Santa Casa de São Paulo São Paulo SP Brasil Faculdade de Ciências Médicas da Santa Casa de São Paulo, São Paulo, SP – Brasil

**Keywords:** Betacoronavirus/fisiologia, COVID-19/metabolismo, Pneumonia, Viral/transmissão, Não - HDL-C, Aterosclerose, Doença Inflamatória, Lipoproteínas LDL/metabolismo

O estudo “Valor prognóstico do colesterol não HDL na pneumonia por COVID-19” avaliou o valor preditivo do colesterol de lipoproteína não de alta densidade (não-HDL-C) em pacientes com SARS-CoV-2 para mortalidade na infecção por Covid-19. Os autores incluíram 1.435 pacientes entre janeiro de 2020 e junho de 2022. Os resultados mostraram que idade, proteína C-reativa e DHL estavam positivamente correlacionados com não HDL-C, e o grupo não sobrevivente era mais velho.^1^ Esses resultados corroboram os resultados dos estudos que demonstram que vários biomarcadores poderiam ser usados como valor preditivo para prognóstico e mortalidade na doença Covid-19.^2^

O não-HDL-C representa uma carga total de lipoproteínas aterogênicas, como LDL-C, VLDL-C, IDL-C, Lp(a) e remanescente de quilomícron.^3^ Pode ser usado como um preditor de risco, mesmo em amostras sem jejum, quando os triglicerídeos aumentam. Por outro lado, as infecções por COVID-19 diminuem os níveis de colesterol total, LDL-C, HDL-C e apolipoproteína AI, A-II e B, enquanto os níveis de triglicerídeos podem estar normais ou aumentados. O grau de redução do colesterol total, LDL-C, HDL-C e apolipoproteína AI poderia ser preditivo de mortalidade. Níveis reduzidos de HDL-C e apolipoproteína AI avaliados antes de infecções por COVID-19 estariam associados a um risco aumentado de infecções graves por Covid-19, enquanto níveis de LDL-C, apolipoproteína B, Lp (a) e triglicerídeos não estariam. As infecções por Covid-19 alteram os níveis de lipídios e lipoproteínas, e os níveis de HDL-C podem afetar o risco de desenvolver infecções por Covid-19.^4^

Curiosamente, a carga aterosclerótica de não-HDL-C estaria ligada a doenças inflamatórias e hipertrigliceridemia.^5^ Além disso, várias doenças inflamatórias crônicas (síndrome metabólica, psoríase, vírus da imunodeficiência humana, hepatoesteatose não alcoólica e síndrome da apneia obstrutiva do sono) estariam associadas à aterosclerose subclínica e maior risco cardiovascular.^6 - 11^ Vale ressaltar que os baixos níveis de HDL-C estão relacionados observacionalmente e geneticamente a riscos aumentados de doenças infecciosas, morte durante sepse, diabetes mellitus e doença renal crônica. Dados observacionais indicam associações de baixo HDL-C com várias doenças autoimunes, cânceres e mortalidade por todas as causas.^12^ A relação entre não-HDL-C e Covid-19 parece existir.

Curiosamente, doenças autoimunes, como a psoríase, também foram associadas a níveis mais altos de enzima conversora de angiotensina tipo 2 (ECA2) do que a população em geral. Observou-se que a proteína spike Covid-19 tem uma alta afinidade pelos receptores ECA2. Este poderia ser um possível mecanismo causal de reatividade na associação entre psoríase e infecção por Covid-19. É possível que o estado hiperinflamatório induzido pelo Covid-19 cause uma regulação positiva de citocinas previamente controladas, desmascarando uma predisposição genética para a psoríase, e queo tratamento com medicação sistêmica antipsoriásica direcionada não necessariamente reduz esse risco.^13^

O não-HDL-C pode ser um marcador precoce de disfunção endotelial vascular em pacientes com diabetes tipo 2.^14^ Além disso, a infecção por SARS-CoV-2 foi associada a um maior risco de diabetes. A infecção por Covid pode mais do que triplicar a chance de ser diagnosticado com diabetes tipo 2 dentro de um ano após a infecção. As pessoas hospitalizadas para tratamento da Covid tiveram mais do que o dobro do risco de serem diagnosticadas com diabetes tipo 2, e as internadas em unidades de terapia intensiva tiveram mais do que o triplo do risco.^15^

O estado inflamatório tem sido associado a um maior risco residual cardiovascular em comparação com o estado aterosclerótico em pacientes recebendo tratamento clínico otimizado. Nesse contexto, os pacientes podem apresentar níveis mais elevados de mediadores inflamatórios (TNF-alfa, IL-1, IL-6). Recentemente, pesquisadores demonstraram que em pacientes recebendo estatinas contemporâneas, a inflamação avaliada pela proteína C-reativa de alta sensibilidade foi um preditor mais forte para o risco de eventos cardiovasculares futuros e morte do que o colesterol avaliado pelo LDL-C.^16^

O processo inflamatório estaria ligado a um maior risco cardiovascular, explicando que pacientes com doenças de alta carga inflamatória evoluem com maior gravidade e risco de mortalidade. Mais atenção deveria ser dada à inflamação sistêmica para fornecer melhores estratégias preventivas.

Novas pesquisas demonstram que uma ferramenta de estratificação para pacientes de alto risco cardiovascular pode ser testada para uma melhor estratificação. O índice imunoinflamatório sistêmico (SII), que é derivado das contagens de neutrófilos, plaquetas e linfócitos, representa o equilíbrio homeostático entre o estado inflamatório, imunológico e trombótico. O SII tem sido relatado como um novo marcador prognóstico em tumores e doenças cardiovasculares. É um marcador de risco, barato e de aplicação simples. O SII esteve intimamente associado à morte cardiovascular e à morte por todas as causas.^17^ Em outro estudo, o SII foi um preditor independente de eventos adversos maiores em pacientes com IAMCSST e pode ser usado para melhorar a predição de eventos adversos, especialmente quando combinado com fatores de risco tradicionais.^18^

Recentemente, os resultados do estudo CLEAR OUTCOMES mostraram que o ácido bempedóico (BA), um inibidor da ATP citrato liase, baixou o LDL-C, mas também os níveis de proteína C-reativa de alta sensibilidade. Este mecanismo subjacente aos potenciais efeitos anti-inflamatórios do BA é incerto.^19^

Em resumo, pesquisas futuras revelarão mais ligações entre doenças inflamatórias associadas a doenças cardiovasculares e cardiometabólicas. Todas essas descobertas, tanto diagnósticas quanto terapêuticas, provavelmente ajudarão a reduzir as comorbidades e as taxas de mortalidade, especialmente em pacientes mais graves.

Os autores do estudo^1^ devem ser parabenizados pela pesquisa interessante e atual e pelos resultados obtidos.
