# Supplementary material for: Relação Metabólica e Inflamatória entre Covid-19 e Não-HDL-C
Source: Arq Bras Cardiol. 2023 Jun 16;120(6):e20230304. [Article in Portuguese] doi: 10.36660/abc.20230304 (PMC10344079; doi:10.36660/abc.20230304)
Supplement: Translation

# Translation: Metabolic and Inflammatory Relationship between Covid-19 and Non-HDL-C

**Authors:** Renato Jorge Alves

**Affiliations:** 1 Brazil Irmandade da Santa Casa de Misericórdia de São Paulo, São Paulo, SP – Brazil; 2 Brazil Faculdade de Ciências Médicas da Santa Casa de São Paulo, São Paulo, SP – Brazil

**Keywords:** Betacoronavirus/physiology, COVID-19/metabolism, Viral, Pneumonia/transmission, Non-HDL-C, Atherosclerosis, Inflammatory Disease, Lipoproteins LDL/metabolism

The study “Prognostic Value of Non-HDL Cholesterol in COVID-19 Pneumonia” evaluated the predictive value of non-high-density lipoprotein cholesterol (non-HDL-C) in patients with SARS-CoV-2 for mortality in Covid-19 infection. The authors included 1435 patients between January 2020 and June 2022. The results showed that age, CRP, and LDH were positively correlated with non-HDL-C, and the non-surviving group was older.^1^ These results corroborate studies’ results demonstrating that several biomarkers could be used as predictive value for prognosis and mortality in Covid-19 disease.^2^

The non-HDL-C represents a total burden of atherogenic lipoproteins, such as LDL-C, VLDL-C, IDL-C, Lp(a), and chylomicron remnant.^3^ İt may be used as a predicted risk, even in nonfasting samples when triglycerides increase. On the other hand, COVID-19 infections decrease total cholesterol, LDL-C, HDL-C, and apolipoprotein A-I, A-II, and B levels, while triglyceride levels may be normal or increased. The degree of reduction in total cholesterol, LDL-C, HDL-C, and apolipoprotein A-I could be predictive of mortality. Decreased HDL-C and apolipoprotein A-I levels evaluated before COVID-19 infections would be associated with an increased risk of severe Covid-19 infections, while LDL-C, apolipoprotein B, Lp (a), and triglyceride levels would not be. Covid-19 infections alter lipid and lipoprotein levels, and HDL-C levels may affect the risk of developing Covid-19 infections.^4^

Interestly, the atherosclerotic burden of non-HDL-C would be linked to inflammatory diseases and hypertriglyceridemia.^5^ Moreover, several chronic inflammatory diseases (metabolic syndrome, psoriasis, human immunodeficiency virus, non-alcoholic hepatosteatosis, and obstructive sleep apnea syndrome) would be linked to subclinical atherosclerosis and higher cardiovascular risk.^6 - 11^ It is worth emphasizing that low HDL-C levels are related observationally and genetically to increased risks of infectious diseases, death during sepsis, diabetes mellitus, and chronic kidney disease. Observational data indicate associations of low HDL-C with various autoimmune diseases, cancers, and all-cause mortality.^12^ The relationship between non-HDL-C and Covid-19 seems to exist.

Interestly, autoimmune disease such as Psoriasis has also been associated with higher levels of angiotensin-converting enzyme type 2 (ACE2) than the general population. Covid-19 spike protein has been noted to have a high affinity for ACE2 receptors. This could be a possible causal mechanism of reactivity in the association between psoriasis and Covid-19 infection. It is possible that the hyper-inflammatory state induced by Covid-19 causes an upregulation of previously controlled cytokines, unmasking a genetic predisposition for psoriasis, and that treatment with targeted anti-psoriatic systemic medication does not necessarily mitigate this risk.^13^

The non-HDL-C may be an early marker of vascular endothelial dysfunction in patients with type 2 diabetes.^14^ Furthermore, SARS-CoV-2 infection was associated with a higher risk of diabetes. The Covid infection can more than triple the chance of being diagnosed with type 2 diabetes within a year of being infected. People hospitalized for Covid treatment had more than a doubled risk of being diagnosed with type 2diabetes, and those admitted to intensive care units had more than a tripled risk.^15^

The inflammatory status has been liked to a higher cardiovascular residual risk compared to atherosclerotic status in patients receiving optimal clinical treatment. İn this context, the patients may show higher inflammatory mediators levels (TNF-alpha, IL-1, IL-6). Recently, researchers demonstrated that in patients receiving contemporary statins, the inflammation assessed by high-sensitivity CRP was a stronger predictor for risk of future cardiovascular events and death than cholesterol assessed by LDL-C.^16^

The inflammatory process would be linked to a higher cardiovascular risk, explaining that patients with diseases with a high inflammatory load evolve with greater severity and risk of mortality. More attention should be paid to systemic inflammation to provide better preventive strategies.

New research demonstrates that a stratification tool for high cardiovascular-risk patients could be tested for better stratification. The Systemic immune-inflammatory index (SII), which is derived from neutrophil, platelet, and lymphocyte counts, represents the homeostatic balance among inflammatory, immune, and thrombotic status. The SII has been reported as a new prognostic marker in tumors and cardiovascular diseases. İt is a risk marker, not expensive, and a simple application. The SII was closely associated with cardiovascular death and all-cause death.^17^ In another study, the SII was an independent predictor of major adverse events in patients with STEMI and may be used to improve the prediction of adverse events, especially when combined with traditional risk factors.^18^

Recently, results of the CLEAR OUTCOMES Study showed that bempedoic acid (BA), an inhibitor of ATP citrate lyase, lowered LDL-C, but high-sensitivity C-reactive protein levels too. This mechanism underlying the potential anti-inflammatory effects of BA is uncertain.^19^

In summary, further research will uncover more links between inflammatory diseases associated with cardiovascular and cardiometabolic diseases. All these discoveries, both diagnostic and therapeutic, will likely help to reduce comorbidities and mortality rates, especially in more severe patients.

The authors of the study “Prognostic Value Of Non-HDLCholesterol In COVID-19 Pneumonia” should be congratulated for the interesting and current research and the results obtained.
